# Novel Protein Sources for Applications in Meat-Alternative Products—Insight and Challenges

**DOI:** 10.3390/foods11070957

**Published:** 2022-03-25

**Authors:** Marcin A. Kurek, Anna Onopiuk, Ewelina Pogorzelska-Nowicka, Arkadiusz Szpicer, Magdalena Zalewska, Andrzej Półtorak

**Affiliations:** Department of Technique and Food Development, Institute of Human Nutrition Sciences, Warsaw University of Life Sciences, Nowoursynowska 159c Street 32, 02-776 Warsaw, Poland; anna_onopiuk@sggw.edu.pl (A.O.); ewelina_pogorzelska_nowicka@sggw.edu.pl (E.P.-N.); arkadiusz_szpicer@sggw.edu.pl (A.S.); magdalena_zalewska@sggw.edu.pl (M.Z.); andrzej_poltorak@sggw.edu.pl (A.P.)

**Keywords:** protein, meat analog, texture, insect protein, algae protein

## Abstract

Many people are increasingly interested in a vegetarian or vegan diet. Looking at the research and the available options in the market, there are two generations of products based on typical proteins, such as soy or gluten, and newer generation proteins, such as peas or faba beans, or even proteins based on previously used feed proteins. In the review, we present the characteristics of several proteins that can be consumed as alternatives to first-generation proteins used in vegan foods. In the following part of the work, we describe the research in which novel protein sources were used in terms of the product they are used for. The paper describes protein sources such as cereal proteins, oilseeds proteins coming from the cakes after oil pressing, and novel sources such as algae, insects, and fungus for use in meat analog products. Technological processes that can make non-animal proteins similar to meat are also discussed, as well as the challenges faced by technologists working in the field of vegan products.

## 1. Introduction

As consumer awareness of the environmental impact of food production increases, so does the consumption of products derived entirely from plants. This has to do with the narrative that meat production requires extensive land and water resources, negatively impacting the terrestrial and aquatic biodiversity and emitting greenhouse gases [1]. More and more people are also avoiding plant-based products, which are very interesting in terms of nutritional value, vitamins, micro and macro elements, and the ability to counteract some of the diseases prevailing among the civilization, due to their cholesterol and blood-pressure lowering properties [2]. This state of affairs influences the substantial growth of the meat analog market, which today is increasing expansively.

Most meat analogs are based on two proteins: soy and gluten. Soy protein is a good alternative to meat in terms of amino acid composition and textural properties [3]. However, the use of soy is quite controversial among consumers due to genetically modified (GM) crops. There is a study showing that 55% of consumers are opposed to GM foods and nearly 60% do not trust GM scientists [4]. Some consumers are strongly against GM soy application in feed for animals [5]. In contrast, the use of gluten ensures that a proper network is built in the product, but it is a fairly high allergenic raw material and may be avoided by some people [6]. This is because more people are diagnosed with celiac disease as well as gluten intolerance. Although important scientific advances have been made in the understanding of the pathologic mechanisms behind nonceliac gluten sensitivity, this disorder is still a matter of active debate in the scientific community [7]. More and more importance is being given to searching for alternative novel protein sources that can be used in meat analog products.

It is also worth noting that the cost of producing plant protein is significantly lower than the cost of producing animal protein. Of course, this translates into technological properties, but there are a number of methods that try to prevent this, such as protein texturization [8]. According to World Bank reports, there is an increase in demand for animal protein that cannot be met, hence the need for more intensive work on new sources of protein [9].

In 2020, the market value of plant-based-meat worldwide was estimated to be worth USD 6.67 billion. This figure is estimated to steadily increase over the next few years and reach roughly 16.7 billion in 2026. This is also influenced by the opinion on the safety of animal-based proteins, which are linked to epidemics such as mad cow disease, swine influenza, or avian flu that appear from time to time [10]. For this reason, this literature review is a systematization of knowledge in the utilization of novel protein sources according to origin—plant, microbial, fungal, insect, and algae.

## 2. The Function of Protein in Meat-Alternative Products

Proteins play an important role in human nutrition by providing building materials essential for both growth and cell regeneration. First of all, most of the meat alternative food sources from which proteins can be obtained differ in the composition of proteins themselves, as well as their amino acid profiles. Most often, meat alternatives do not use only protein isolates; concentrates or powders derived from plants, such as dietary fiber, vegetable fat, or carbohydrates, may also be included in the formulation. For many years, plant protein was considered to have lesser nutritional value, but this trend is now reversing [11].

Concerning nutritional values, it is worth noting that plant protein is not consumed as an individual ingredient but in a group with other ingredients. Therefore, it is not easy to control the potential effects of different nutrients from different meat alternative foods and attribute the observed benefits solely to the protein content. Furthermore, specific sources of plant and animal protein in the diet have been shown to have different health effects. Thus, general statements about plant or animal protein may be too simplistic, and effects may depend on the dietary matrix and accompanying nutrients.

Regarding nutritional properties, it is worth focusing on the fact that the meat analog has similar or comparable nutritional value to an identical meat-based product. As a rule of thumb, if a product has more than 30% protein with low-fat content, it can be considered a good meat substitute [12]. At the same time, it is also worth noting that substitutes or alternatives to meat products should be characterized by their similarity to meat protein digestibility-corrected amino acid score (PDCAAS) [13]. Supplementation or obtaining iron or vitamin B_12_ from other sources is crucial if meat is excluded from the diet entirely.

Protein has a number of technological functions specific to a particular protein origin and degree of concentration—depending on whether it is a formulation, concentrate, or isolate. These characteristics include solubility, thermal stability, emulsification, flavor binding capacity, and digestibility score [14]. These characteristics are directly linked to the technological and functional roles in creating meat analog products.

From a technological point of view, it is not really possible to create a direct alternative to meat protein solely from the plant-based protein (except for the use of cultured meat target) product. This is due to aspects such as the reconstruction of the fiber network, which would reflect the myofilaments that are crucial for shaping tenderness and juiciness. Therefore, product development research on plant-based alternatives has largely been limited to restructured (or reconstructed) products [15]. This makes the alternative meat products mainly belong to two groups ranked by particle size. These can be divided into coarse-particle products, such as burgers, patties, sausage, meatballs, nuggets, etc., and fine-particle products, which are highly homogenized products that often have emulsion properties.

Many of the proteins are used in meat applications as ingredients due to their properties of functioning as a water binding agent. Several proteins are often combined together for better results, such as pea protein isolate–wheat gluten blends and soy protein isolate–wheat gluten blends [16]. In terms of whole meat protein substitution, soy protein, which consists of albumin and globulin fractions, is the most common. In addition to soy protein, pea protein is also common. Most proteins derived from legumes possess the gel-forming ability, which is crucial because viscoelastic gel structure plays a major role in adhering particles, immobilizing fat, and entrapping water within the matrix of emulsion-type alternative protein products [17].

A widely used protein in meat alternative applications is gluten, which imparts the appropriate chewiness to products. The elasticity and extensibility properties of gluten are ascribed to two major protein fractions: glutenins and gliadins, which specifically influence the structure of meat products [18].

However, it is worth noting that soy is often associated negatively among consumers due to its strong association with genetic modification. At the same time, gluten is a highly allergenic protein and is not tolerated by people struggling with celiac disease.

## 3. Protein Sources and Their Roles in Meat-Alternative Products

### 3.1. Legume Proteins

In recent years, consumers have paid particular attention to plant-based diets. This is due to the increasing public awareness of the health-promoting effects of bioactive compounds from plants on human health and the willingness to reduce meat consumption for environmental reasons [19]. Of particular importance in the diet are legumes, whose effect on inhibiting diseases has been scientifically confirmed. These plants belong to the group of annual plants of the Fabaceae family of legumes [20]. Their edible part is the fruit, or the so-called pods, which are eaten whole or partially depending on the species and the degree of maturity of the fruit. The seeds of leguminous plants are characterized by their high nutritional value. Compared to other plants, they are distinguished by a fairly high protein content, ranging from 20% to 35% on average, depending on the type, growing conditions, and degree of maturity. Legume seeds are a rich source of dietary fiber, vitamins, minerals such as magnesium, iron, zinc, potassium, and phosphorus, and compounds with high antioxidant potential [21]. The seeds of these plants are low in saturated fats and, like all plant foods, are free from cholesterol [22,23]. A legume-rich diet improves bowel function and benefits hormonal balance [24]. Legume seed protein differs from cereal grain protein in amino acid composition—a significantly higher proportion of lysine (especially peas) and threonine, whereas the factor limiting its biological value is the insufficient content of sulphur amino acids (methionine and cystine) and tryptophan. In the protein of legume seeds, two fractions are distinguished: albumin and globulin. Albumins make up 10–25% of the total protein, can be soluble in water, and are mainly found in the germinal part. They are structural and enzymatic proteins, forming complex linkages with carbohydrates, lipids, and nucleic acids. The more albumin a seed contains, the greater its nutritional value. Globulins are soluble in dilute solutions of neutral salts. Different legume species provide varying amounts and qualities of protein to organisms [25]. Edible legume species include peas, lentils, lupins, chickpeas, broad beans, and mung beans.

Peas are an excellent source of protein and are exceptionally high in lysine and threonine, as well as other essential amino acids. They have a low glycemic index. Numerous scientific studies show that peas play a large role in preventing colon cancer and help treat breast cancer, pancreatic cancer, prostate cancer, lung cancer, and leukemia. Lentil seeds are also full of nutritional value because they contain 9/100 g protein and 0.4/100 g fat in edible parts; they are rich in iron, phosphorus, magnesium, and B vitamins. There are many types of lentils, including red, brown, green, yellow, and black lentils, among others [26].

Lupin seeds are another type of legume protein source whose nutritional value of low-alkaloid varieties is comparable to soybeans. Among legumes, lupin seeds contain the most protein (up to 46%) and the least undesirable non-nutrients. Due to the presence of functional components, they have potent health-promoting properties. They show antioxidant and hypocholesterolemic activity, have a low glycemic index, increase the bioavailability of minerals, and have anti-allergic and anti-inflammatory effects [27]. Lupine seeds, as well as soybeans, can be used in the production of both traditional and functional foods. 

Proteins from other legumes are also used in the production of meat analogs. In recent years, many studies have been conducted on the possibility of using chickpea, faba bean, and mung bean proteins in the production of meat analogs [28]. One of these is the study by Bühler et al., in which the researchers subjected faba bean protein concentrate to heating [29]. This led to modifications in the water holding capacity and solubility of the protein, achieving properties similar to soy protein concentrate, which is used in most meat analogs. This study showed that the choice of ingredients for meat analogs should depend both on the protein content and source and its nutritional value, but also on its thermal processing history, which can have a decisive influence on its technological properties. Among the previously noted three species of legumes, chickpea is the most consumed by consumers [30]. Chickpea protein is characterized by good properties in terms of texture, ability to bind water and oil, and ability to gel. The ability to stabilize emulsions and foam comparable to soy protein isolate and whey proteins is also an important property of chickpea protein. Moreover, chickpea protein isolate shows the ability to absorb more fat and a similar amount of water compared to soy protein isolate [31]. An essential advantage of chickpea is its positive effect on the color of the meat analog. Studies have shown that partial replacement of textured vegetable protein with chickpea flour significantly increased the color acceptability of meatless nuggets. The reason for this is the carotenoids contained in chickpeas [32].

Faba bean proteins may be a promising ingredient for producing meat analogs. Like chickpea proteins, they are excellent in stabilizing emulsions and foam, but to a lesser extent than soy protein isolate [32]. This is a limiting factor for using faba bean proteins as an ingredient in meat analogs. Many factors influence the technological utility of plant proteins. Thus, it is possible to improve the functionality of legume proteins as a result of appropriately selected parameters of production and processing processes. The study showed that dry fractionation enhanced the properties of protein-rich faba bean flour compared to faba bean protein isolate produced by acid extraction. Dry fractionation produced proteins with higher solubility at pH 7. The gelling and foaming abilities were also improved [28]. Faba bean proteins have been successfully used in the production of meat analogs by wet spinning, shear cell technology, and high moisture extrusion methods [33].

Mung bean proteins are also growing in popularity as an ingredient in meat analogs. The mung bean is a plant valued for both its nutritional value and functional properties. It is characterized by high protein (25–28%) and low fat content (1–2%). A significant amount of proline, glutamic acid, arginine, leucine, and phenylalanine is present in mung bean protein [34]. The limiting amino acid in mung bean protein is leucine. Notably, the digestible indispensable amino acid score (DIAAS) for this protein is 86, compared to 91 for soybean protein and 70 for pea protein. Mung bean protein is composed mostly of globular proteins, resulting in good gelling properties [35]. Like chickpea and faba bean proteins, mung bean proteins show the ability to stabilize foams and emulsions. Mung bean proteins are, therefore, used to obtain a balanced amino acid profile and desirable textural properties of meat analogs because they have globulins (60%, vicilin-type 8S with MW 26–60 kDa), albumins (25%, MW 24 kDa), and other globulins including basic-type 7S and legumin-type11S [17,34].

### 3.2. Oilseeds Proteins

In recent years, many oilseeds have been used as sources of protein in the food industry. The whole seeds and meals obtained from them are a valuable source of proteins with a well-balanced profile of essential amino acids with sulphur-containing amino acids. Their antioxidant, antihypertensive, and neuroprotective properties make them a valuable and functional alternative source of protein, e.g., in the baking and meat industry. The oil plants used as a source of protein include, inter alia, soybean, chia seeds, evening primrose, flaxseed (brown), hemp seeds, milk thistle, nigella seeds, pumpkin seeds, rapeseed, sesame, safflower, glandless cottonseed, and sunflower seeds [36].

In addition, these proteins complement desirable functional properties when added to certain foods; this applies to whipping capacity, viscosity, emulsifying capacity, and water and oil binding capacity. Rapeseed and soybean protein isolates have a higher whipping capacity than sunflower, peanut, sesame, cottonseed, and safflower. Furthermore, the addition of sugar improves the whipping properties of the oilseed proteins. In contrast, if the oilseed proteins are heated, the whipping ability is reduced. Of the oilseed proteins, soy protein has the greatest emulsifying power. The emulsifying properties of heat-treated oilseed proteins are similar or better than that of animal proteins. Cotton seed protein has a very high water and oil binding capacity. However, the water-binding capacity of the oilseed proteins gradually decreases with increasing heating time at 100 °C. In contrast, heated oilseed proteins have an oil binding capacity similar to or better than that of unheated proteins [37]. In addition, the low allergenicity of pumpkin and hemp seeds or the potential non-allergy of evening primrose, milk thistle, black cumin, and chia compared to legume proteins makes it possible to use them as functional ingredients in newly developed food products [36].

Among the many benefits of proteins obtained from oil plant seeds, one should also remember the dangers of plants such as rapeseed that contain, in addition to many nutrients beneficial for the human diet, toxic erucic acid and sulfur compounds—glucosinolates, which are a component of the protein fraction [38]. To reduce the amount of anti-nutritive compounds (including glucosinolates, sinapin, and phytic acid) from proteins derived from oilseeds, innovative extraction methods are used.

### 3.3. Cereal and Pseudocereal Proteins

In grain-based proteins used in meat analogs, wheat, oats, or rice are used. The most common is wheat protein, which is gluten, due to its viscoelastic properties [1]. Other wheat proteins are not as often used as a base for creating meat alternative products, but due to their properties, they are fairly well distributed as structuring agents, even in true meat products. 

A study conducted by de Angelis et al., indicated that oat protein isolates produced a rather good sensory effect when combined with pea protein [39]. However, the positive sensory properties were only observed after the extrusion process, which positively affected quenching the pea odor. The oats themselves were regarded by consumers for the pleasant smell but still far from being meaty. 

Both legumes and cereals proteins contain pretty significant amounts of phytic acid, which is judged to be anti-nutritional by being strongly element restrictive. Some researchers have additionally introduced enzymatic activity and fermentation to reduce the phytic acid content of meat analog extrudates. The results were quite promising, but too much enzyme activity can end up degrading macromolecules, thus making it difficult to maintain an appropriate texture [40].

A very promising raw material for creating meat analogs is rice, an established low allergenicity raw material and, in particular, is presented as an alternative to soy. Raw rice was reported to be more allergenic than cooked rice, even though some allergens are heat stable and proteolysis resistant [41]. Currently, rice flour is being used as a substitute for fat while taking advantage of its water-binding properties in meat products. The use of 4–6% rice flour effectively increases the firmness of sausage-type meat products while being highly acceptable to consumers [42].

Cereals that are high in protein are pseudo-cereals like amaranthus and quinoa. Amaranth and quinoa grains are equally good as cereals and legume seeds because of their high content of lysine, arginine, tryptophan, and other sulphur-containing amino acids. Amaranth is an example of a plant with a high protein content of up to 14%. Some difficulty in obtaining protein is the isolation of starch in the case of amaranth [43]. Amaranth itself also has a flavor that consumers may not fully accept. However, amaranth has already been successfully used as a binding agent in sausage formulations. 

Another type of pseudo-cereal used in meat products is quinoa, a raw material with approximately 8% protein but a very high nutritional value containing all nine essential amino acids. The use of quinoa in meat products improved its water-holding capacity, reduced its toughness, and positively affected the sensory experience [44,45]. Further work on protein concentration and isolation from quinoa may lead to a good base combination for creating legume-based meat analogs.

When using grain-based proteins, they must be proteins with a fairly good amino acid profile. At the same time, existing concentrates or formulations have a widely accepted flavor and are not treated negatively. The ability to bind water means that in the future, they can be used as additional proteins in the composite to create meat analogs [30].

### 3.4. Algae Proteins

Algae, or photosynthetic eukaryotes, are distinguished as microalgae and seaweed. Microalgae is a huge group covering almost 200,000 species [46]. Out of this group, several species have been tested for a variety of purposes: food additives, cosmeceuticals, animal feed, or wastewater treatment. Foods obtained or formulated with the addition of algae are included in the definition of novel foods in the Novel Foods Regulation (EU). Microalgae is a promising novel ingredient that might be applied in the formulation of meat analogs. The growth rate of microalgae cultivars is superior to other crops used as sources of plant proteins. The estimated microalgae yield of dry biomass reaches 15–30 tonnes annually per unit area compared to 1.5–3.0 tonnes for soybeans. Microalgae and seaweed also contain more protein per unit area (4–15 tonnes/Ha per year and 2.5–7.5 tonnes/HA per year, respectively) in comparison to soybean (0.6–1.2 tonnes/Ha per year) or wheat (1.1 tonnes/Ha per year) [47]. Depending on strain and cultivation conditions, microalgae can produce up to 70% of proteins in cells compared to 30–40% for soybeans. Even more important from the quantity of protein occurring in algae is its quality. The nutritional quality of protein is determined by the composition of amino acids and the amount of essential amino acids. Two most dominant microalgae species on the market, Spirulina (*Arthrospira*) and Chlorella, are characterized by the higher than standard (100) essential amino acids index (102.6 and 107.5, respectively). Those values are similar to casin milk protein and higher than soybean meal [48]. There are microalgae of good essential amino acids (EAAs) balance. For instance, *Chlorella* contains 7 EAAs, comparable to beef but with a slightly lower level of cysteine and methionine. However, in most algae species, lysine and tryptophan are limiting amino acids [49]. Further, for brown algae except for the two noted above, also lysine, while for red species, leucine and isoleucine occur in low concentrations. In the case of seaweed, cysteine is most limiting, whereas glutamic acid and aspartic acid are most abundant [50].

Proteins acquired from microalgae exhibit techno-functional potentials such as high solubility and capacity to emulsify and form gels and foam. Solubility of *Chlorella protothecoides* proteins at pH 2–12 is estimated to be approximately 84.3%. For comparison, soybean protein (glycin) at pH 4.5–6.0 is soluble at less than 20% [51]. In turn, emulsifying and foaming are comparable to soy and whey proteins. Some species, such as *Chlorella vulgaris*, have even higher emulsifying properties. Algae proteins are also considered to be safe as food components. Those properties drew the scientific community’s attention towards using algae proteins as a substitute for animal protein. Palanisamy et al., (2019) observed that adding *Spirulina* (*Arthospira platensis*) flour at a level of 30% to lupin protein-based meat decreased in vitro protein digestibility from 82% to 76.5%. However, it was reversed partially by changing the process parameters [52]. Based on the data, the authors stated that *Spirulina* increased nutritional (higher antioxidant activity and phenolic content) and physico-chemical properties of the meat analog. Other studies revealed that adding spirulina at higher concentrations gives the product dark color, musty odor, and intensive earthy flavor [53]. Nonetheless, also in this study, setting the proper process conditions—low moisture content with high temperature and screw speed—enables partly replacing soy with spirulina in meat substitute and obtaining a product of decent flavor quality. Even though methods to produce microalgae rich in proteins on a large scale were invented about 50 years ago, still today, there exists only a few novel products formulated based on them. There are several reasons for that. First of all, algae dry matter contains 10% of the cellulosic cell wall, which is not digested and utilized by humans and non-ruminant animals. Thus, it is required to use various extraction and purification methods, thus increasing the costs of microalgae biomass application and limiting its use to high-value industries. Furthermore, algal protein concentrates are characterized by green and yellow colors and an unattractive fishy odor. Those attributes negatively influence consumers’ perception of meat analogs formulated with algae addition. Sensory experiments showed that the product acceptance decreases with the increase in algae content [54]. Lowering prices was the only way to make eating meat substitutes with algae content more attractive [53]. Some researchers suggest that familiarity with food influences buying behavior and that algae meat analogs are unattractive for consumers because they are still unfamiliar to them. Nonentheless, to date, there is a lack of ideas for how to positively affect consumers’ attitudes toward algae meat substitutes.

### 3.5. Insect Proteins

Insects are common food for 2 billion people in 119 countries across the globe [55]. There are over 2000 edible species. The most consumed insects that are used as protein sources are Coleopatra Beetles (31%), Lepidoptera Caterpillars (18%), Hemynoptera, wasps, bees, and ants (14%). However, those are still novel foods for Western countries. This is slowly changing due to growing need for alternative sources of proteins, production of which would be more sustainable. Studies on the life cycle assessment of Hermetia illucens performed by Smetana et al., (2019) revealed that insect protein concentrates had a lower environmental footprint than animal proteins but higher than plant proteins [56]. In accordance with studies conducted by Mason et al., (2018), the production of one gram of beef requires 21 times more water (16.8 g) than the production of the same amount of protein from cricket (0.7–0.8 g) [57].

Insects are a good source of proteins. The average content of proteins in them is 40% and ranges from 20% up to 70% depending on the species. Three species that are widely bred in Europe (Tenebrio molitor, Gryllodes sigillatus, Schisocerca gregaria) are considered to have the biggest potential as food components in the European Union and contain 52.35, 70, and 76% of proteins, respectively [58]. The amount and quality of proteins within the same species vary greatly depending on diet, metamorphic stage, or habitat. However, protein content is also often overestimated due to the presence in insects of a non-protein nitrogen. It has been estimated that up to 26% of whole larvae nitrogen may be non-protein [59]. Insect proteins are more digestible (76–98%) than plant proteins (lentils 52%) and slightly less digestible than animal proteins (95% egg protein, 98% beef protein) [60]. The essential amino acids’ score for insects ranges from 46% to 96%, which greatly exceeds the lowest recommended level for human diets (>40%). The quantity of the same amino acids is even higher in insects than those from plant and animal protein sources [61]. Insect proteins have high threonine and lysine content but low levels of methionine or tryptophan. Proteins acquired from insects are characterized by a low level of solubility ranging from 3% to 45%. However, the solubility may be improved by enzymatic hydrolysis. For instance, the major solubility improvement of migratory locust protein was observed to be 10–22% and up to 55%. Along with solubility, authors also observed higher emulsifying activity of approximately 54%, enhanced foam ability of approximately 326%, and improved oil banding capacity [62]. Thus, the application of insect proteins is recommended for foods that do not require high solubility, such as meat analogs. Furthermore, insect proteins are especially recommended as an addition to plant meat analogs to improve its protein profile. Smetana et al., observed that using the highest temperature of a barrel extruder (170 °C) made it possible to introduce 40% of insect protein to a soy-based meat analog, keeping its optimal meat-like texture [63]. In turn, Kim et al., (2022) performed studies on the usage of insect proteins along with textured vegetable proteins to produce restructured jerky analogs [64]. In conclusion, the authors of the studies stated that it is possible to produce meat analog combining both of those proteins to get tender jerky of high nutritional value. There is also a study aiming to partially replace meat protein (10%) with insect flour (*Tenebio molitor* or *Bombyx mori*). The results of this experiment indicate that even though they obtained high-value emulsion sausages, those were harder than control meat samples. However, consumer safety is also an issue. There is a risk of an allergic reaction after consuming insect proteins, which contain tropomyosine and arginine kinase—two major proteins responsible for allergic reactions. Furthermore, insect-derived food and feed might be contaminated chemically with heavy metals and biologically with spore-forming bacteria [65].

### 3.6. Edible Fungus Proteins

Mushrooms have been classified into a separate kingdom because of their different cellular organization, and they do not belong to either animals or plants [66]. Fresh edible fungus has about 90% water, and the remaining 10% dry matter is composed of 8–40% protein, 3–28% carbohydrate, 3–32% fiber, 2–8% fat, and 8–10% ash, varying with the mushroom species and other factors [67]. Yu et al., (2020) examined 23 edible mushrooms and determined their protein content. It was found that the protein content in edible mushrooms was approximately 8.5–36.9%, which was much higher than that of vegetables, fruits, and grains. The higher protein content was found in *Trichloma* (36.87%), and Tremella had the lowest protein content (8.46%). Other more popular mushrooms like *Shiitake*, *Lentinus Edodes*, *Volvariella Volvacea*, and *Boletus* had a protein content of 15.38%, 11.59%, 10.24%, and 12.16%, respectively. Fungus proteins are gaining more and more popularity all over the world. As meat production has a significant impact on the environment, it is important to find a cheap, alternative, and less resource-intensive source of protein to partially replace meat or meat products. Mushrooms cannot be considered as a significant source of proteins compared to meat sources, even though they are a part of human nutrition mainly because of their taste [68]. Other authors, however, believe that mushrooms may play an important role in meat analogs by providing nutrients and promoting the development of sensory properties such as appearance, texture, and taste of the product [69]. The use of mushrooms as an alternative source of protein in the human diet is not a new concept. Edible mushrooms can be treated as a functional food due to their nutritional value. The use of edible mushrooms has been used in meat products as meat substitutes or fillers to improve the physicochemical and sensory characteristics and their nutritional value. The production of mycoprotein products is based on submerged fermentation of fungi in a liquid culture medium [70]. The mycoprotein production is based on the continuous fermentation of the filamentous fungus *Fusarium venenatum* on a glucose substrate, which allows the production of a high-protein, low-fat food ingredient [71]. They are usually grown in bioreactors with a high metabolic rate. Miller and Dwyer (2001) assessed the tolerance of humans to mycoprotein, and the results demonstrated that mycoprotein is well tolerated by humans and has an extremely low allergenic potential [72]. Singh et al., (2021) indicate that the mycoprotein of some fungi is a good source of protein. Still, due to its low digestibility, it is rarely used to prepare meat analogs, although *Fusarium venenatum* is cultivated to derive mycoprotein and prepare meat substitutes [1]. The mycoprotein may have a meat-like texture and flavor. Some researchers argue that proteins produced using mycoproteins have structures similar to muscle fibers of meat and claim that mycoproteins can be considered as an alternative source of the food protein. Due to their functional properties, it is possible to use them in new attractive health-promoting food products. The use of biotechnological methods for their production creates an opportunity to reduce production costs and improve the sensory and nutritional properties [73]. The harvested mycoprotein can be used to prepare vegetarian sausages, burger patties, or minced cutlets. Other mushroom-based meat substitutes are produced from *Aspergillus oryzae*. Filamentous mushrooms are used in most mushroom-based meat products because their long fibers create a meat-like texture. Denny et al., (2008) stated that the mycoprotein may have a meat-like texture and flavor and is the main component of various mycoprotein products, including minced meat, chicken pieces, burgers, sausages, nuggets, fillets, ready to eat meals, cakes, and pies [71]. In many Asian countries, *Monascus purpureus*—treated with yeast produce red rice—and *Aspergillus oryzae*—fermented with soy—is used in hamanato, miso, and shoyu. Nowadays, in the European market, Quorn™, a meat substitute originated in Great Britain, is sold. Quorn™ contain mycoprotein derived from the *Fusarium venenatum* filamentous fungus [74]. Mushrooms and fermented products have a meaty taste, a long shelf life, good nutritional values, and reduced cooking time, so they can be a new generation of plant proteins in the future. All real products in which the novel sources of proteins were used are summarized in Table 1.

## 4. Processing of Proteins Applied in Meat-Alternative Products

**Figure 1:**
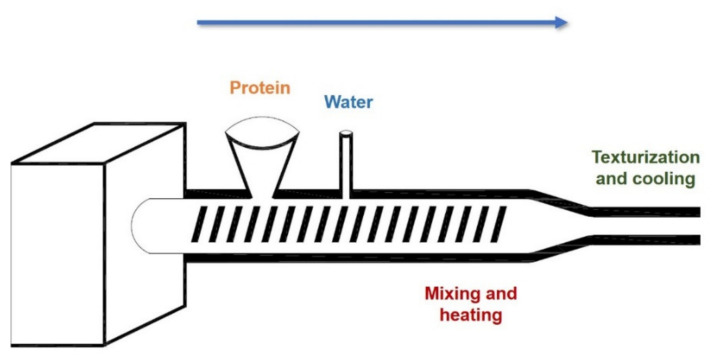
Process of texturization of proteins for application in meat analog production.

Textured vegetable protein (TVP) was one of the first ingredients used in the production of meat analogs. The TVP production technology was developed in the 1970s, and it was then that this type of product was introduced to the market for the first time. Initially, TVP was used as a filler in various conventional food products. In the following years, the development and production technology of meat substitutes based on TVP began. The primary raw material for TVP production is soy proteins, although other ingredients such as cotton, wheat, and corn are also used. Nevertheless, the TVP consists mainly of processed dried soy flour to give it a spongy texture and is flavored to improve the meat-like sensory properties. TVP is produced in the extrusion process (Figure 1). High-temperature (120–200 °C) and high-pressure (20 MPa) processing of the raw material make it possible to obtain products of various shapes (such as cubes or stripes), sizes, colors, and textures [17,75].

In the 1980s, the fiber spinning technique began to be used to produce meat analogs. In this method, the alkaline protein solution was forced through the matrices into the acidic coagulation base. This led to the precipitation of fibers that were mixed with bonding materials. However, the process was very complex, required a highly concentrated protein solution, and had lower yields than large-scale production costs [76].

Currently, the main technology for producing this type of product is thermoplastic extrusion. Extrusion used in recent years is a method characterized by high efficiency and allows for the reduction of the energy cost of production. Skimmed vegetable proteins are made with the addition of water, salts, carbohydrates, lipids, flavors, and other functional additives. The mass is then put on the extruder screw where the product structure is shaped under the influence of high temperature and pressure [77].

**Table 1:** Summary of real products where the novel sources of proteins were applied.

Type of Proteins	Source of Protein	Type of Product	Characteristic Traits	Reference
Legume	Faba bean	Texturized product after high-moisture extrusion (HME)	The best parameters of HME: 130 and 140 °C, water:product ratio = 4 and feed rate 11 rpm (1.10 Kg/h), good bite-feeling, good elasticity/firmness, positive sensory attributes	[78]
	Mung bean	Texturized product after extrusion cooking	Optimized extrusion parameters: 49.33% feed moisture, 80.66 rpm screw speed and 144.57 °C barrel temperature, partial protein unfoldment, fibrous structure, high retention of amino acids	[34]
Oilseeds	Soy protein Isolate–gluten	Couette cell product	More layered and fibrous structured products, formation of anisotropic structures in the microscale	[79]
	Lima bean and African oil bean seed	Texturized vegetable protein (TVP)	Higher overall acceptance than cooked meat, Concentrations of essential amino-acids range between 0.90 and 7.3% with a near absence of anti-nutritional factors (0.0022–1.0008) g/kg	[75]
Cereal and pseudocereal	Pea protein dry-fractionated, pea protein isolated, soy protein isolated and oat protein	Extrudates from twin-screw extruders	Lower water absorbtion for samples with oat protein; intense odor and taste profile for samples with pea protein dry-fractionated and oat protein	[39]
	Oat protein concentrate and pea protein isolate	Texturized product after extrusion cooking	Extruded product with minimum recommended amounts of essential amino acids for adults but lower content of phytic acid 1.5%	[40]
	Rice flour	Meat-based sausages	Lower cooking loss and better emulsion stability for the samples with rice flour	[42]
	Black quinoa	Bologna-type sausage	Better emulsion stability, lower water activity and lipid oxidation values	[44]
Algae	Spirulina platensis flour	Lupin protein based meat analogs	Improved physico-chemical and nutritional properties	[52]
	Spirulina	Spirulina-soy extrudate for pasta filling	Decreased liking of product with higher content of soy-spirulina filling	[54]
Insects	*Alphitobius diaperinus*	Insect based meat analog	Hardness texture and protein composition similar to meat	[63]
	Mealworms	Restructured jerkey analog	Similar texture and nutrient quality to animal meat	[64]
Edible fungus proteins	Filamentous fungus *Fusarium venenatum*	Quorn^TM^ meat substitute or cooking ingredient	A meat-like texture and flavour, high-fibre, low-fat food ingredient, an average protein content of 45%	[71,74]
	*Aspergillus oryzae* fermented with the soybean	used in hamanato, miso, and shoyu	5–10% protein content, meaty flavour, long-shelf life	[1]
	*Lentinus edodes*, *Coprinus comatus* and *Pleurotus ostreatus*	Mushroom-based meat sausage Analog	Texture and flavour close to beef, a satisfactory level of consumer acceptability	[69]

To meet the expectations of customers, the production of meat analogs focuses on obtaining acceptable sensory characteristics such as taste, smell, color, and consistency. Two methods of extracting proteins used for the production of meat analogs are known as ‘dry’ and ‘wet’ extrusion. Unfortunately, “dry extrusion” (humidity <30%) does not allow for obtaining a sensorially acceptable product. In contrast, ‘wet extrusion’ (humidity 40–80%) enables the production of meat analogs of premium quality. The preparations obtained through this method are characterized by a consistency resembling real meat, and their appearance and mouthfeel are similar to cooked meat [52]. Due to the use of high moisture extrusion (HME), it is possible to produce from raw materials with low solubility, and in addition, this method is more economically viable [80]. Meat analogs produced by HME from soy protein are the most common [78], but using this method, it is possible to obtain high-moisture meat analogs (HMMAs) from plants, such as hemp, yellow pea, lentils, and faba bean [64,81,82].

The latest technical solutions are based on Couette thermostatic shear, in this case, the suspension of proteins and gluten gels in a linear flow. Because of this process, it is possible to obtain a product characterized by a fibrous structure. Moreover, shear-induced structuring with a high-temperature shear matrix created fibrous protein structures. The developed closed-chamber rheometer allows you to control thermal and mechanical stresses. Due to this, it is possible to obtain conditions similar to extrusion [79].

## 5. Challenges for Protein Applications in Meat-Alternative Products

Although the current development trend is towards developing foods for vegetarians, almost every product has similar challenges. These are not only technological but also consumer or even sociological challenges.

From a sensory point of view, achieving a viable alternative to a meat product is quite difficult because the specificity of meat in terms of amino acid structure, peptide sequences, and intermolecular connections is very specific and impossible to counterfeit. Sensory properties and, in particular, mouthfeel are influenced by a texture with very low granularity that is able to bind water. In order to maintain these characteristics, plant proteins must be subjected to several different structuring processes, such as thermomechanical extrusion or shear. Despite the use of a number of methods that alter the structure of plant proteins or increase water-holding capacity, there are still many difficulties that need to be solved. One of them is juiciness, which is a specific characteristic of meat, resulting from water absorption and the form of water-binding with proteins and in their fibers. Hence there is currently no possibility to replace meat with proteins of the same or similar texture.

Although plant proteins are the most common alternative to meat proteins, they have a particular taste that is different from meat. For example, in legume-protein products, an aftertaste derived from a characteristic beany odor is thought to be related to the secondary lipid oxidation products [83].

In addition to texture and palatability issues, meat products are characterized by a red, reddish, or pink color that, for obvious reasons, is impossible to achieve without the use of colorants. Unfortunately, the problem is present because many consumers interested in vegetarian products are consumers who avoid additives, which further increases the technological difficulty [84]. It is the lack of a clean label that makes consumers uncomfortable with meat protein product alternatives. Vegetarian products that are alternatives to meat protein products often contain a very high amount of ingredients like preservatives, stabilizers, colorants, or thickeners [17].

The protein alternative must also be a nutritional alternative, which is understood by adequate nutrient density. Unfortunately, because protein source alternative products are highly processed products, their nutritional value is not the same as meat products produced directly from raw meat. This is mainly because the protein used to produce the alternatives is already processed by heat and other methods. There is still no clear confirmation whether replacing meat protein with vegetable protein does not negatively impact human health by reducing the supply of heme protein, zinc, or selenium, which are characteristic of products based directly on meat. Meat processing methods and meat alternatives such as grilling, roasting, frying, and baking are considered methods that can lead to increased concentrations of carcinogenic substances such as heterocyclic aromatic amines [85]. However, using polyphenolic substances in plant-based products is easier than adding to meat products, which may lead to reduced HAA formation [19].

## 6. Conclusions

Today, more and more consumers are turning to vegetarianism or looking for products that are not based on animal products. This is understandable from the point of view of worldview, religion, or often just the search for new tastes. In most meat analogs, we encounter proteins of soy origin and wheat origin, like gluten. Unfortunately, both of these proteins are allergenic, and additionally, soy is associated with GMO crops, which are also negatively perceived by some people. The development of a range of meat analog products is possible by using novel sources of protein as well as methods of processing. These can be raw materials rich in protein, such as legumes, or by-products of various processes, as in the case of oilseeds cakes. Novel sources of protein are algae, insects, and fungus. With texturization technology, it is possible to obtain a product of sufficient quality in terms of texture. At the same time, it is worth bearing in mind that it is almost impossible to obtain the texture of meat, so analogs can only be suitable analogs of meat products after processing.

The increase in demand for plant-based protein will certainly be seen in future years as we look for new sources of protein to meet the needs of a growing population. In developed countries, more consumers are turning to vegetarianism and veganism, which will also contribute significantly to the demand for such products. However, a certain unmatched element will be the elaboration of not only the nutritional, but more importantly the physical and technological properties that meat protein possesses. Some hope is offered by zoonotic sources such as insects and from single-celled organisms such as algae because of their easy modification.

## Data Availability

No new data were created or analyzed in this study. Data sharing is not applicable to this article.
